# Supplementary figures and images for: Full-length transcriptome analysis of Adiantum flabellulatum gametophyte
Source: PeerJ. 2022 Mar 9;10:e13079. doi: 10.7717/peerj.13079 (PMC8917799; doi:10.7717/peerj.13079)

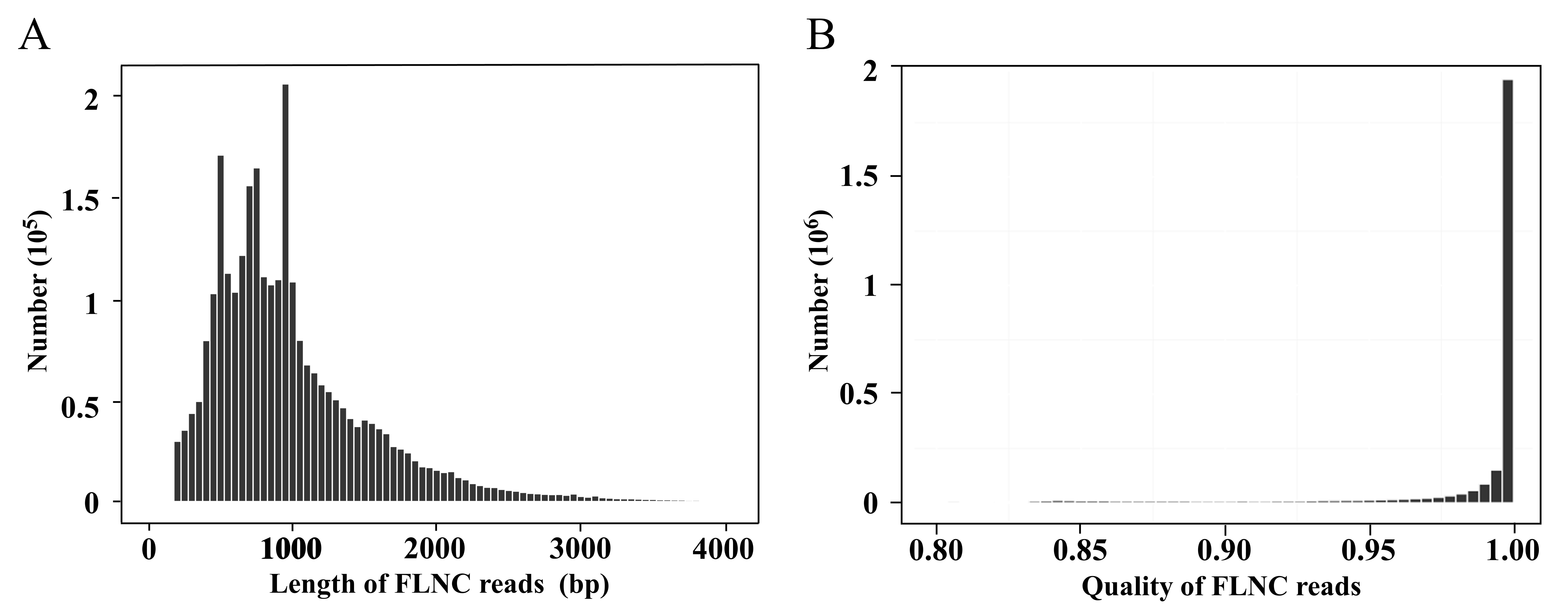

Supplement: Supplemental Information 12 — (A) Length distributions of FLNC reads; (B) quality distributions of FLNC reads. [file peerj-10-13079-s012.png]

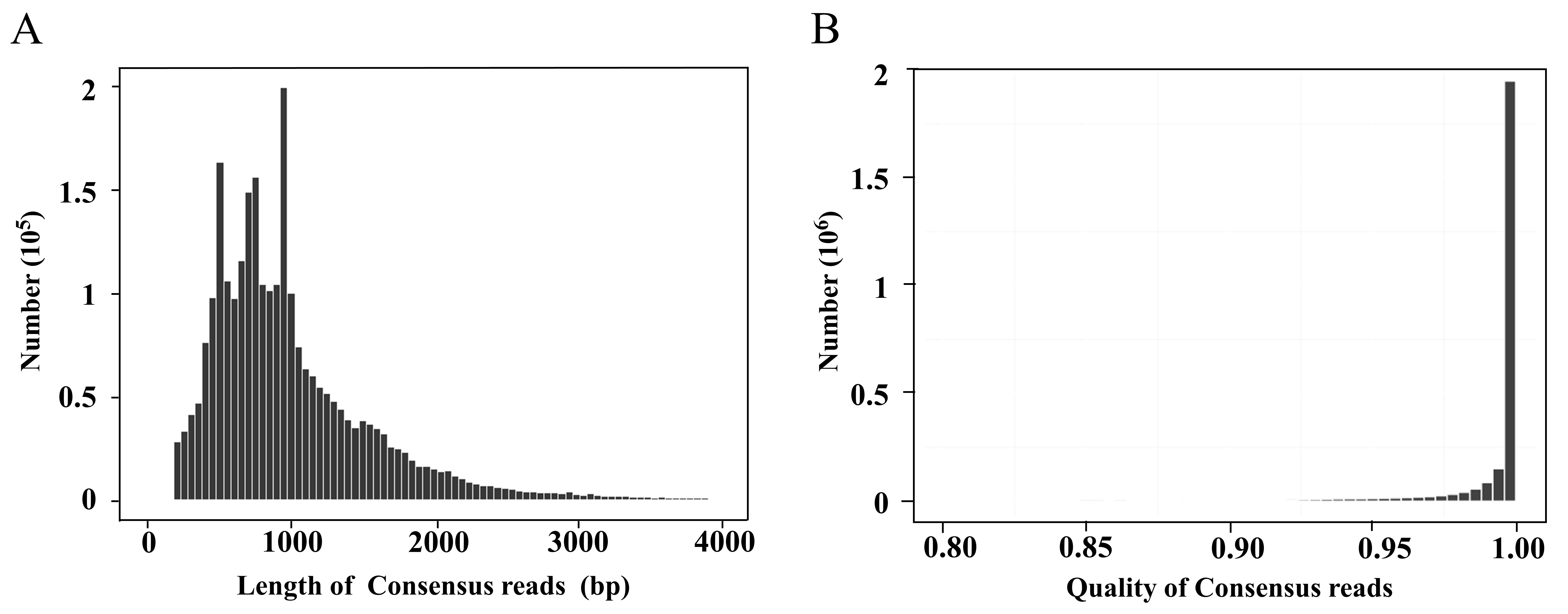

Supplement: Supplemental Information 13 — (A) Length distributions of consensus reads; (B) quality distributions of consensus reads. [file peerj-10-13079-s013.png]
